# Deciduous Molars Complexity Anatomy Reveled by Computed Microtomography

**DOI:** 10.1055/s-0043-1772566

**Published:** 2023-09-20

**Authors:** Tais Fernandes Teixeira, Alexandre Marques Paes da Silva, Thais Machado de Carvalho Coutinho, Eduardo Fagury Videira Marceliano, Ana Raquel Lopes dos Santos Miranda, Dennis de Carvalho Ferreira, José Claudio Provenzano, Marilia Fagury Videira Marceliano-Alves

**Affiliations:** 1 Postgraduate Program in Dentistry, Estácio de Sá University, Rio de Janeiro, Brazil; 2 Department of Endodontic Research, Iguaçu University, Nova Iguaçu, Rio de Janeiro; 3 Dental Clinic Department, Brazilian Army General Hospital of Belem, Belem, Brazil; 4 Department of Dentistry, University Center of Pará, Belém, Brazil

**Keywords:** pediatric dentistry, deciduous teeth, dental anomalies, radiology

## Abstract

**Objective** The aim of this study was to analyze the internal morphology of deciduous molars through the use of computed microtomography in a sample from Rio de Janeiro.

**Material and Methods** Thirty maxillary and 30 mandibular deciduous molars ( *n* = 60), divided in first and second primary molars, were scanned by computed microtomography. The teeth were evaluated for root number, root canals, Vertucci classification, root curvature, presence of lateral canals, furcation dentin thickness, structure model index (SMI), volume, and canal surface area.

**Results** The results showed 100% of maxillary molars had three roots and Vertucci type I canal was more prevalent in this group. In the mandibular ones, type IV was more frequent in the mesial root and class I in the distal root and the cavo-interradicular canal occurred in 2 specimens. Dentin thickness in the furcation region measured 1.53 and 1.59 mm in the maxillary and mandibular, respectively. Volume and area parameters varied according to the evaluated canals and SMI demonstrated that all canals had a cylindrical shape.

**Conclusion** More detailed information about the internal anatomy of the primary molars has been described, which may help strategies in the preparation of these root canals.

## Introduction

The premature loss of deciduous teeth can have consequences on the permanent dentition, such as producing changes in the eruption guide of the permanent tooth, phonetic disorders, and aesthetic commitment, among others. 1 The deciduous teeth exhibit anatomical differences when compared with permanent ones in terms of size, external, and internal morphology. These have a thin layer of mineralized hard tissue (enamel and dentin) between the external and internal surfaces, which can lead to rapid pulpal involvement due to carious lesions. As a consequence, these deciduous teeth are subject to endodontic treatment. 2

Among all the factors that should be considered for achieving success in endodontic therapy in deciduous teeth, the professional needs to know the internal anatomy. Thus, the same should keep in mind the common characteristics and possible anatomical variations of the root canal system (RCS) of this dentition, to obtain a better cleaning, disinfection, and modeling of the RCS. 3

As in the permanent dentition, in the decidua many factors can influence the failure of the endodontic treatment. It should be noted that among the possible causes of failure in this treatment modality in deciduous teeth, the limitations are the process of performing the technique properly since there are difficulties such as the definition of the apical limit of the canal due to physiological and inflammatory resorptions, persistent infections, behavioral conditioning of the child, as well as aspects related to the anatomy. These factors require greater attention in mechanical chemical preparation and the use of antimicrobial effects. 4 5 Studies show that deciduous molars are the teeth most affected by cavities, consequently, they may be more susceptible to pulp involvement. 6

In the current literature, only one study addressing detailed anatomical data of primary molar teeth through the use of computerized microtomography (micro-CT) has been performed to date. Thus, this study had the objective of adding qualitative and quantitative data of maxillary and mandibular deciduous molars through a nondestructive and high-resolution technology.

## Material and Methods

### Sample Selection

This study was previously submitted and approved by the Research Ethics Committee (61593416.2.0000.5284). For the study, 60 maxillary and mandibular deciduous human molars were selected, extracted for reasons unrelated to this study, from the Institutional Teeth Bank. The teeth included in the study should have up to one-third of one of the roots resorbed and without previous endodontic treatment. Molars that presented incomplete rhizogenesis, reabsorption of more than one-third of the root, and with root fractures were excluded.

### Microtomographic Examination and Assessment of the Internal Anatomy

To acquire the images, the teeth were scanned in the SkyScan 1174 device (Bruker Micro-CT, Kontich, Belgium), 19.9 µm, with 1304 × 1024 pixel (small pixel size) and radiation source of 50 Kv, 800 µA, and aluminum filter 0.5 mm thick. After confirming the position, the projections of the specimen were initiated at various angles, during a 180-degree rotation, with rotation steps of 1.0 degree. The scanning time was approximately 17 minutes per specimen.

Then, reconstruction of the three-dimensional (3D) images of the specimens was performed in the program NRecon 1.6.6.0 (Bruker Micro-CT) with the correction and reduction of artifacts (beam hardening correction of 5.0%, ring artifact reduction of 5, and smoothing 5). The CTAn 1.1.4.1 (Bruker Micro-CT) program allowed quantitative data acquisition and creation of 3D models, which were visualized in the program CTvol 2.3.2.0 (Bruker Micro-CT) for qualitative evaluation.

### Qualitative Evaluation

The roots were classified by Vertucci's classification, 7 and the direction of curvature, number of canals per root, presence and location of lateral canals, as well as interroot canals were also recorded after visualizing the 3D images created in the CTVol software. The angle of curvature was measured in the ImageJ program (National Institutions of Health, Bethesda, Maryland, United States) according to the method of Schneider. 8

### Quantitative Analysis

In the CTAn (Bruker Micro-CT) program, the dentine thickness was evaluated between the pulp chamber floor and the external portion of the furcation by means of a linear measurement tool. Other 3D data were evaluated: volume, area, and structure model index (SMI), which is used to evaluate the shape of the canal, following the parameters: SMI value close to zero the canal is flat; the value close to 3 is classified as cylindrical; and when close to 4 is considered a sphere.

## Results

A total of 60 deciduous molars were included in the present study and were distributed in four groups (mandibular first molars, mandibular second molars, maxillary first molars, and maxillary second molars), each group containing 15 elements and the quantitative and qualitative data are described as follows.

**Fig. 1:**
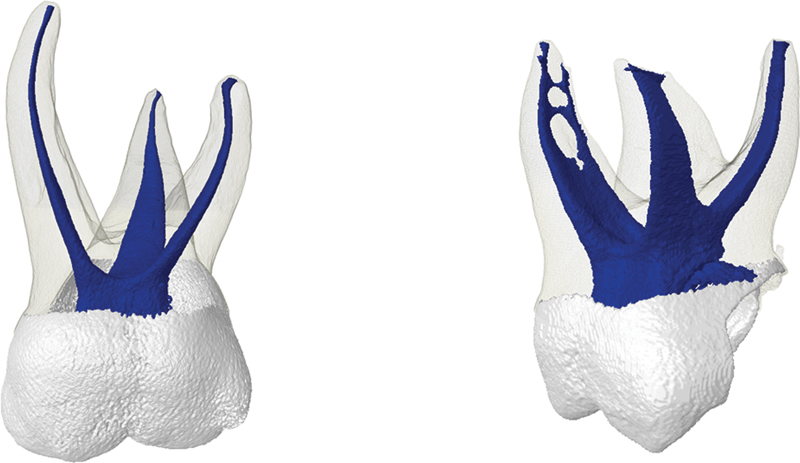
Representative images of deciduous maxillary molars in the present study.

**Fig. 2:**
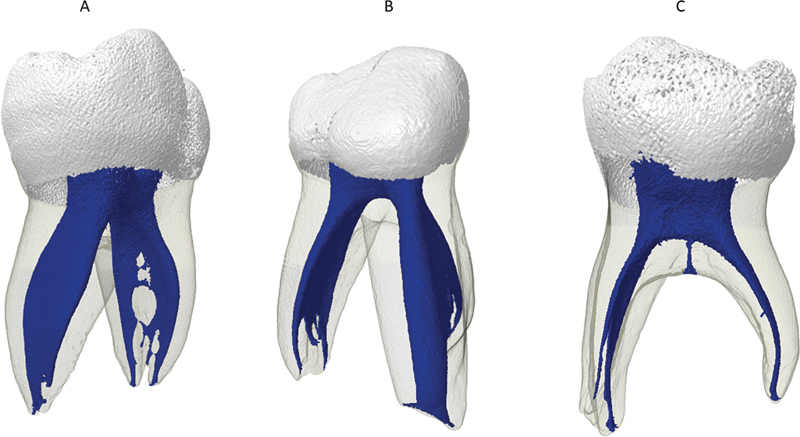
Representative images of deciduous mandibular molars in the present study. ( **A** ) Vertucci's type I and IV. ( **B** ) Vertucci's type V. ( **C** ) Interradicular *cavus* canal.

In the qualitative analyses of the external anatomy, all the maxillary molars had three roots (mesiobuccal, distobuccal, and palatine) and all mandibular molars had two roots (mesial and distal). The first and second maxillary molars presented more frequently in all Vertucci type I roots ( Table 1 and Fig. 1 ). In the mesial roots of mandibular first molars, 80% of type IV or V were found, and type I was the most prevalent in the distal one ( Fig. 1 ). In the mandibular second molars, the mesial root presented type V in 46.5% of the cases, and the distal root in 93% of the samples were class I or II ( Table 1 and Fig. 2A and B ).

**Table 1:** Morphology of the root canals of the maxillary and mandibular deciduous molars, according to the Vertucci classification

	Mandibular				Maxillary					
Vertucci's classification	Primary molars		Secondary molars		Primary molars			Secondary molars		
	Mesial	Distal	Mesial	Distal	Mesiovestibular % ( *n* )	Distovestibular	Palatine	Mesiovestibular % ( *n* )	Disto vestibular	Palatine
	% ( *n* )	% ( *n* )	% ( *n* )	% ( *n* )		% ( *n* )	% ( *n* )		% ( *n* )	% ( *n* )
Class I	13 (2)	60 (9)	13 (2)	46.5 (7)	93 (14)	100 (15)	100 (15)	84 (13)	93 (14)	100 (15)
Class II	–	7 (1)	–	46,5 (7)	–	–	–	–	7 (1)	–
Class III	–	–	13 (2)	–	7 (1)	–	–	7 (1)	–	–
Class IV	40 (6)	33 (5)	20.5 (3)	7 (1)	–	–	–	–	–	–
Class V	40 (6)	–	46.5 (7)	–	–	–	–	7 (1)	–	–
Class VI	7 (1)	–	7 (1)	–	–	–	–	–	–	–

In only two specimens the presence of the interradicular *cavus* canal was detected, both in the mandibular molars ( Table 2 and Fig. 2C ). A total of 22 lateral canals were detected in the mandibular first molars and 19 of these canals were located in the middle thirds ( *n* = 11) and/or apical ( *n* = 8), while in the mandibular second molars 19 lateral canals were identified as apical. In the maxillary ones, 12 lateral canals (only 2 in the apical third) were visualized in the first molars and 17 in the second molars (11 in the apical third of the canals).

**Table 2:** 3D morphometric results of root canals of mandibular primary molars revealed by computerized microtomography

Tooth	Canal	Average ± SD		
		Volume (mm ^3^ )	Area (mm ^2^ )	SMI
	Mesial singular	23.36 ± 2.77	72.26 ± 4.26	2.12 ± 1.17
	Mesiovestibular	3.80 ± 1.23	27.10 ± 7.08	2.24 ± 0.40
	Mesiolingual	3.04 ± 1.55	21.56 ± 7.79	2.05 ± 0.40
First mandibular molar	Distal singular	7.44 ± 5.67	36.32 ± 9.75	1.75 ± 0.45
	Distovestibular	3.38 ± 1.25	23.08 ± 6.91	2.37 ± 0.30
	Distolingual	3.38 ± 1.17	24.46 ± 6.42	2.13 ± 0.26
	Cavo-interradicular canal	0.03	1.29	2.45
	Mesial singular	8.95 ± 1.91	62.50 ± 9.63	1.21 ± 0.07
	Mesiovestibular	3.41 ± 1.19	24.98 ± 7.04	2.28 ± 0.57
	Mesiolingual	2.71 ± 1.53	23.25 ± 8.52	1.98 ± 0.40
Second mandibular molar	Distal singular	5.38 ± 2.26	37.30 ± 7.74	1.41 ± 0.30
	Distovestibular	5.89 ± 0.59	23.50 ± 2.63	1.91 ± 0.26
	Distolingual	6.15 ± 1.58	22.06 ± 1.69	2.14 ± 0.17
	Cavo-interradicular canal	0.06	0.50	2.33
	Mesiovestibular	3.17 ± 1.06	26.86 ± 8.37	2.41 ± 0.71
First maxillary molar	Mesiolingual	0.84	9.02	2.35
	Distovestibular	3.32 ± 2.13	25.50 ± 9.73	2.70 ± 0.75
	Palatine	3.32 ± 2.41	31.15 ± 7.61	2.70 ± 0.65
	Mesiovestibular	4.54 ± 2.66	28.87 ± 7.82	3.02 ± 0.78
Second maxillary molar	Mesiolingual	0.47 ± 0.29	7.61 ± 4.64	2.61 ± 0.16
	Distovestibular	5.13 ± 2.43	26.13 ± 9.24	2.96 ± 0.81
	Distolingual	1.17	11.54	2.57
	Palatine	7.12 ± 3.77	31.93 ± 7.27	3.59 ± 1.71

Abbreviations: 3D, three-dimensional; SD, standard deviation; SMI, structure model index.

In the quantitative analyses the degree of curvature of the mandibular molars of the mesial canals showed a mean angle of curvature of 35.39° occurring in the distal direction and the distal canals also presented a mean of 36.12° in the mesial direction. In the mandibular second molars, both mesial and distal canals presented mean curvatures of 37.53 and 34.54°, respectively, toward furcation. The maxillary molar curvature of the first molar was 32.57, 32.53, and 27.85° for the mesiobuccal, distobuccal, and palatine roots, respectively, and the mean molar was 35.99, 39.53, and 29° for the same roots in both cases the curvature was directed to furcation. The most voluminous canals were detected in the mesial roots of the mandibular molars (with only one canal in the mesial root, detected in four specimens) and palatal in the mandibular and maxillary molars, respectively ( Table 2 ). The SMI values of the canals of the mandibular and maxillary deciduous molars indicate that, in general, the canals are shaped like a cylinder.

## Discussion

The knowledge of the internal morphology of the RCS becomes imperative for the success of endodontic therapy. 7 In particular, in pediatric dentistry, the most used imaging tests to aid diagnosis and to perform procedures involving deciduous teeth are periapical radiographs. However, these have limited information regarding the size, origin, and location of periradicular and associated lesions, as well as the difficulty of diagnosing inflammatory processes in the early stages and fractures. Finally, another limitation of this radiographic technique is the overlap of anatomical structures. 9

It is known that there are anatomical differences between the deciduous and permanent teeth, both in size and in the internal and external morphology of the same. 10 Some studies have been devoted to describe the morphology of permanent teeth, mainly through the use of nondestructive and high-resolution techniques such as micro-CT, 11 12 but only one study to date has analyzed primary molars using micro-CT analysis. 13

There are several factors that may be associated with the failure of primary endodontic therapy. Among them, the persistence of resistant microorganisms, 14 obturation level, 15 iatrogenies, 16 and presence of poorly adapted restorations. 17 Studies indicate that among these factors, normally, the most associated to failure is the persistence of infection in RCS. 4 5 18 19 20 For other authors, this failure may be associated with untraced canals, and thus remaining untreated, thus allowing the persistence of pathogenic microbiota in recesses or branches. 21 These anatomical variations serve as a protection for the same in the irrigation and modeling procedures of the RCS. 22 23

To confirm the relationship of the microbiota to the failure, a study was performed 4 using scanning electron microscopy (SEM) and histology, with teeth submitted to endodontic treatment that were indicated for extraction due to persistence of clinical signs and symptoms. The authors verified the presence of microorganisms adhered to the canal walls and recess areas, concluding that the cause of the failure was the maintenance of bacteria in RCS irregularities.

A study confirmed that endodontic techniques in deciduous teeth are limited for RCS disinfection and filling, and for this reason, sodium hypochlorite is indicated for irrigation due to the antibacterial spectrum and the propensity for dissolution of necrotic tissues. 24 Another factor that may hamper is the reabsorption that occurs in a physiological way, leading to the formation of perforation in the canal wall and in the furcation, changing the shape, dimension, and position of the apex. 25

It is possible to find in the literature studies that evaluated the morphology of deciduous teeth using different methods, which present advantages, but require the nonreuse of the sample. 26 27 28 29 30 31 32 A viable option to overcome this deficiency is the use of cone-beam computed tomography and micro-CT, which allow the evaluation of several RCS parameters without the destruction of the sample. 5 33 34 35

Micro-CT is a method that has been widely used in endodontic research that analyzes dental morphology. This method allowed the qualitative and quantitative evaluation of the 60 teeth included in this study, all from individuals from the city of Rio de Janeiro, Brazil, as well as from other studies performed in other places (Taiwan and Turkey). 5 34 35 As the anatomical distribution suffers great influence of genetic, 20 this study is justified to compare the anatomical characteristics of the deciduous teeth of individuals from the city of Rio de Janeiro, considering that previously a work has been performed in the southeast region, but in the state of Sao Paulo. 5

These data can be compared with other study that also used micro-CT as a method for the evaluation of deciduous teeth. For the mandibular teeth, the authors found values very different from those of the present study, mainly because although they reported the presence of two canals in the mesial roots in 90% and 55% in the distal of all the specimens, in the quantification of volume, area, and SMI, the authors made the two canals together, which may have masked the result. As for the superiors, the results of the mesiobuccal and palatine canals were similar, whereas the distobuccal ones were found to be smaller (1.65 mm ^3^ , 16.8 mm ^2^ ).

The mean SMI values of the mandibular molar canals of the present work ranged from 1.21 to 2.37 indicating a cylinder shape. As for the superiors, the averages varied from 2.35 to 3.59, indicating a cylindrical geometry. 5 Like the RCS anatomy of the permanent molars, the anatomy of the deciduous molars is considered complex. It is possible to observe the presence of accessory canals, isthmuses, and recesses with frequency, even greater than in the permanent dentition that can be considered within the norms of normality. 5 33 34 35 36 Although they did not classify by the method of Vertucci, 7 several works quantified the number of canals by root. For the purposes of comparison, the data found in this work show that for the mandibular teeth two roots and two canals were found in the mesial roots (85%) and in the distal one 70% presented a canal. In the maxillary ones, three roots were found, and 100% of a single canal was found for both the vestibular and the palatine, while the mesiobuccal root had 95% and the remaining 5% were of two canals, the mesiopalatine canal.

Although using different methods, the number of roots can be compared with the existing data in the literature and this data was consistent with those found in previous studies. 32 33 36 37 A previously performed study demonstrated different results of this study, being verified 50% with three or two roots in the maxillary molars. 35 Regarding the mandibular molars analyzed in the present study, it was possible to observe that 100% of the cases had two roots, one mesial, and one distal, coinciding with previous studies. 32 33 36 As for the number of canals detected, previous studies 2 35 detected in 60% and 80% of the mandibular molars, four root canals, respectively, two in each root. Some authors reported presence of two canals in the mesial root in almost 100% of the evaluated specimens. 32 33 36 39 These differences can be justified by ethnic factors, considering that this study evaluated Brazilian individuals, while the other evaluated Turks. 34 Another factor may be the evaluation method, since micro-CT is a high-resolution evaluation method it can allow evaluation of more detailed RCS parameters. 5 35 39

The peculiar conformation and topography of the root canals in deciduous teeth with sharp curvatures and extensive findings of accessory canals make it difficult to access and instrument these teeth. 36 In relation to the cavo-interradicular canals, only 2 (3.33%) mandibular primary molars were found in the present study. This data was similar to that found in another study, which studied frequency, location, and diameter of these canals in deciduous molars by SEM, finding around 3.5%. 39 Another parameter evaluated in this work related to furcation was the thickness of dentin. The data showed that in the maxillary molars the thickness was 1.53 mm and for the mandibular 1.59 mm. Until the closure of this study, no studies were found that evaluated this parameter; however, this one shows great clinical importance, since the presentation of radiolucent lesion in the furcation region can occur in deciduous molars in cases of necrosis indicating a connection with the periodontium. Therefore, the knowledge of the average thickness and prevalence of canals in this area is essential. 25

Different techniques of endodontic treatment in deciduous teeth are proposed in the literature to promote cleaning and disinfection of the canals. Research has shown that the anatomy of the third apex is meandering and emphasizes that selection 5 of instruments has to be careful and additional methods such as passive ultrasonic irrigation and negative pressure irrigation should be used. 13 16

Other researchers have previously described that endodontic treatment in deciduous teeth presents difficulties due to the peculiar anatomy or even the behavior of the child. 5 When observing the current stage of endodontic treatment in permanent teeth, constantly searching for techniques that optimize the same, we are faced with the need to reevaluate the current stage of endodontic technique in deciduous teeth. Recalling that pulp therapy requires periodic clinical and radiographic follow-up of the treated tooth and support structures in the patient. 13 16 39

## Conclusion

The analysis of deciduous teeth with the use of micro-CT described that the SMI parameter of all canals presented a flattened cylindrical shape, with the exception of the distal canal of the mandibular molars, which were shown in a flat shape. The mandibular molars had a higher frequency configuration of the Vertucci type IV and V canal, while the superior type I was the most observed. This study sought to assist clinicians in understanding the morphological variations of deciduous canals to overcome deficiencies and thus allow appropriate strategies for endodontic treatment, since in pediatric patients it is characterized as a challenge in clinical practice.

## Highlights

This research shows the pediatric dentists more detailed information about the anatomy of deciduous teeth by the use digital microtomography. Knowledge of the internal anatomy of deciduous dentition root canals and their possible variations is of the utmost importance, allowing pediatric dentists to avoid iatrogenic accidents. Another important point that this research can provide is the ability to keep these deciduous teeth in the mouth until their successors erupt. This fact is only possible if pediatric dentists perform endodontic treatment satisfactorily, when these teeth have carious cavities with pulp involvement. For this, it is necessary to know the internal anatomy of the canals and their variations.
